# Immunoproteasome inhibition reduces donor specific antibody production and cardiac allograft vasculopathy in a mouse heart transplantation model

**DOI:** 10.3389/frtra.2024.1494455

**Published:** 2024-12-16

**Authors:** Allison M. Schwalb, Imran Anwar, Isabel DeLaura, Joseph M. Ladowski, Janghoon Yoon, Rafaela Belloni, Mingqing Song, Carolyn Glass, Jun Wang, Stuart Knechtle, Jean Kwun

**Affiliations:** ^1^ Duke Transplant Center, Duke University School of Medicine, Durham, NC, United States; ^2^ Department of Pathology, Duke University School of Medicine, Durham, NC, United States

**Keywords:** immunoproteasome, antibody-mediated rejection, CAV, regulatory T cells, alloantibody

## Abstract

**Objective:**

Cardiac Allograft Vasculopathy (CAV), a process of vascular damage accelerated by antibody-mediated rejection (AMR), is one of the leading causes of cardiac transplant failure. Proteasome inhibitors (PIs) are utilized to treat AMR, however PI-associated toxicity limits their therapeutic utility. Novel immunoproteasome inhibitors (IPIs) have higher specificity for immune cells and have not been investigated for AMR in cardiac transplant patients. We sought to evaluate IPI effect on AMR in a murine cardiac transplant model.

**Methods:**

Fully MHC mismatched C57BL/6 to huCD52Tg heterotopic heart transplantations were performed. Recipients were treated with alemtuzumab (10 µg, IP) on days −2, −1, 2, and 4 and anti-CD25mAb (PC61, 100 µg, IP) on day 7 to accelerate AMR with or without IPI (ONX-0914,15 mg/kg, SQ), administered on transplant day and three times a week thereafter.

**Results:**

Animals without IPI gradually developed post-transplant donor-specific antibody (DSA) and showed a significantly elevated DSA level compared to animals receiving IPI. (TFXM 48.86 vs. 14.17; *p* = 0.0291, BFXM 43.53 vs. 6.114; *p* = 0.0031). Accordingly, H&E staining of allograft showed reduced evidence of AMR with IPI compared to controls (*P* = 0.0410). Notably, increased mortality was observed in the IPI treated group.

**Conclusion:**

This study demonstrated the ability of ONYX-0914, an IPI, to control post-transplant DSA production and the AMR development in a heart transplant model. However, IPI-resistant DSA production was also observed and increased mortality with IPI therapy raises concerns about potential toxicity. Further investigation is warranted to assess the utility and potential risk associated with the use of IPI as a post-transplant maintenance immunosuppression.

## Introduction

Heart transplants provide life-saving treatment for patients with end-stage heart failure. Advances in management of heart transplantation have significantly improved overall survival in the first-year post-transplant; however, there has been little change to the risk of death after 1-year post-transplant (1). The leading cause of heart transplant failure is cardiac allograft vasculopathy (CAV), the development of intimal hyperplasia and vascular fibrosis within the vasculature of transplanted cardiac tissue (2, 3). CAV is a consequence of endothelial damage by both immune and nonimmune factors. and prevention of hyperlipidemia, hypertension, and other non-immune mediated CAV risk factors have been shown to reduce mortality in heart transplant recipients (4). Antibody-mediated rejection (AMR) has been identified as a primary immune-mediated catalyst in the development of CAV (5, 6). Furthermore, episodes of acute AMR and donor-specific antibody (DSA) production have been specifically associated with increased likelihood of CAV development (5, 7) and cardiovascular mortality (8). Preventing and managing AMR is thus paramount to improving graft survival and reducing morbidity and mortality in heart transplant recipients.

Plasma cells secreting antibodies including DSA are important therapeutic targets to address AMR. Currently, most treatment options for AMR are focused either on antibody (plasmapheresis or IVIg) or B cell removal (via rituximab) (9). Proteasome inhibitors (PIs), through their inhibition of the 20s subunit of the proteasome and accumulation of intracellular proteins, preferentially lead to the apoptosis of plasma cells and prevention of antibody production (10, 11). To this effect, PIs such as bortezomib have been utilized clinically to both prevent and treat AMR in solid organ transplant recipients (12). Unfortunately, use of PIs is limited due to their nonspecific inhibition of proteasomes in all cells, resulting in significant toxicity which limits therapeutic dosing and long-term utilization in transplant recipients (13–15).

Novel immunoproteasome inhibitors (IPIs) present an attractive alternative to PIs to manage and prevent CAV. Immunoproteasomes, the target of IPIs, are more highly expressed in cells of hematopoietic origin or those which have been exposed to inflammatory mediators such as IFN-γ and TNF-α (16, 17). IPIs' selective inhibition has been shown to reduce toxicity profiles without compromising plasma cell depletion activity in multiple myeloma (18) and pre-clinical models of solid organ transplant (19). However, the efficacy of immunoproteasome inhibition in preventing AMR has not been evaluated in cardiac transplantation models. Early studies in murine models have suggested that immunoproteasomes are up regulated during acute and chronic AMR in heart transplantation, which validates the potential utility of immunoproteasome inhibition in preventing AMR, DSA production, and CAV (20). The goal of the present study is to determine whether post-transplant IPI treatment can prevent the development of DSA, reduce AMR, and prevent CAV in a murine heart transplant model (21).

## Materials and methods

### Animals

Homozygous huCD52Tg (H-2^k^) mice originally provided by Herman Waldman (22). HuCD52Tg (H-2^k^) mice were bred as homozygotes and maintained at Duke Laboratory Animal Resources. C57BL/6 (H-2^b^) mice were purchased from the Jackson Laboratory (Bar Harbor, ME). The mice were housed in a pathogen-free barrier facility. The study was approved by the Duke University Animal Care and Use Committee (IACUC#A055-21-03).

### Heterotopic heart transplantation

**Figure 1:**
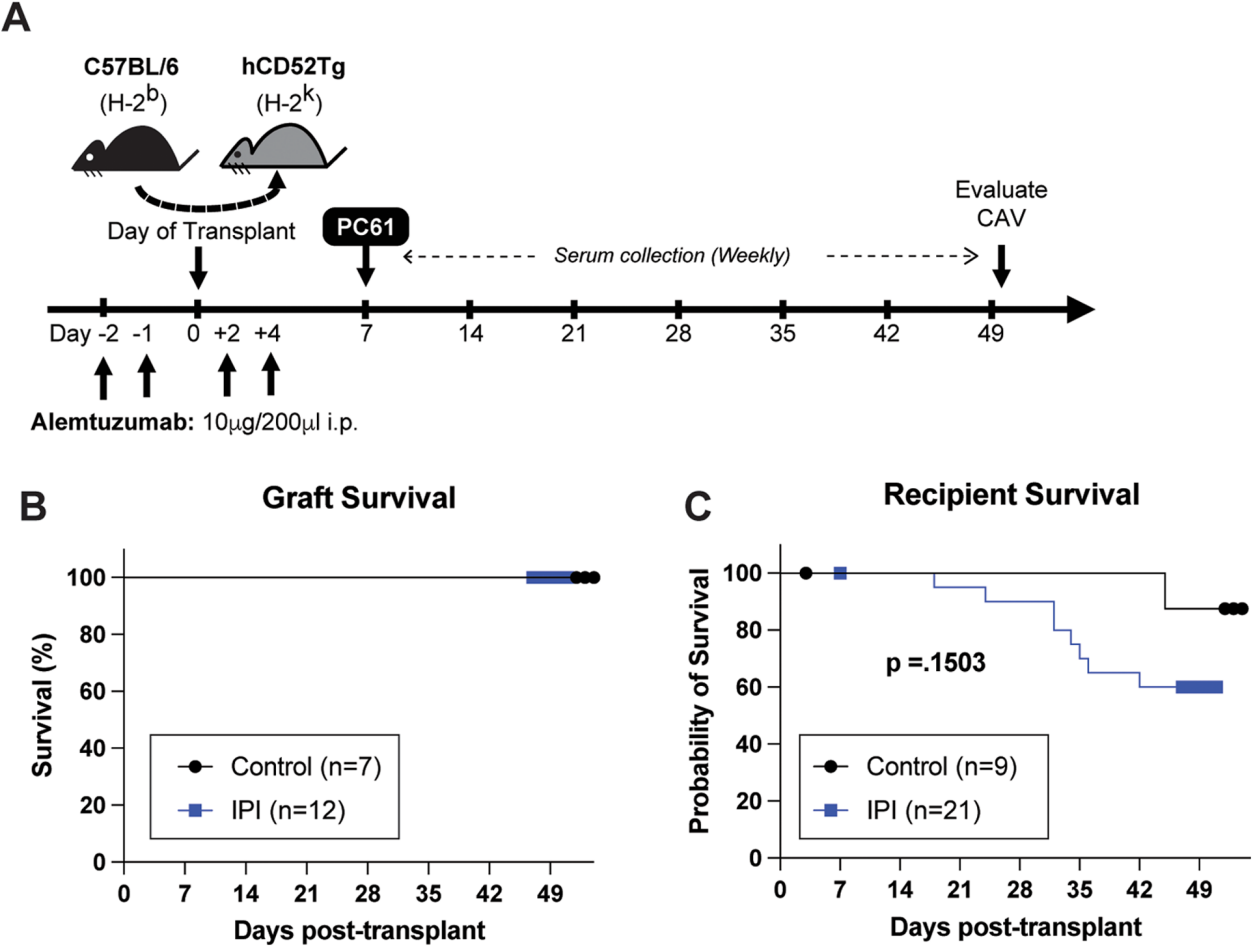
Schematic representation of experimental design and overall survival comparisons between IPI and control groups. **(A)** Visual representation of drug administration and data collection timepoints during the 7-week study period. **(B)** Comparison of heterotopic allograft survival in control group (black) and IPI group (blue) recipients that survived to study completion. **(C)** Comparison of animal survival between recipients assigned to the control and IPI groups during the study period.

At approximately 6–12 weeks of age, the C57BL/6 donor hearts were transplanted into the huCD52Tg recipients using a technique like that described previously (21, 23). The recipients were treated with 10 µg of alemtuzumab (i.p.) at days −2, −1, +2, and +4 relative to transplantation. The recipients also received 100 µg of anti-CD25 mAb (PC61, i.p.) at POD 7 to deplete T regulatory cells and accelerate AMR (24). A total of 30 mice were assigned to one of two treatment protocols: immunoproteasome inhibitor (IPI group, ONX-0914, IV, three times weekly at 15 mg/kg) and control (no injection). Mice in the IPI group were administered IPI between post-transplant days 7 and 50 (Figure 1). All animals were sacrificed at 7 weeks post-transplant.

### DSA detection

A flow cytometry crossmatch was performed to measure DSA as described previously (21, 24). Recipient blood was obtained from the recipients via submandibular bleeding at POD 7, 14, 28, 42, and at the time of sacrifice. Donor splenocytes were prepared from C57BL/6 mice. Briefly, recipient sera were incubated with the donor splenocytes for 20 min at 4°C in the dark. The cells were thoroughly washed and 3 µl of FITC-conjugated anti-mouse Ig (polyclonal; BD biosciences) was added to the samples for a 20 min incubation. The T cells were stained with APC-conjugated anti-CD3 (Clone 145-2C11, BD biosciences) and the B cells were stained with anti-B220 mAb (RA3-6B2; BD biosciences). The samples were analyzed using a BD LSRFortessa X-20 (BD Bioscience, San Jose, CA) and anlayzed using Flow Jo v10.9.0 (Tree Star, San Carlos, CA). Alloantibody production was calculated as median fluorescence intensity fold increase over the negative control (background signal). Non-responders to IPI treatment were defined as mice that demonstrated a 15-fold rise in DSA over background control TFXM during the study period.

### Histology and pathological gradings

The grafts from surviving mice were recovered 7 weeks after transplantation. The explanted grafts were bisected and fixed in 10% formalin or frozen. Sections were stained for H&E and whole stained slide were scanned with an Aperio ScanScope XT (Aperio Technologies, Inc., Vista, CA). Images were assessed by a clinical cardiac transplant pathologist (G.G.) using the ImageScope (Aperio Technologies). A determination of AMR, CAV, and acute cellular rejection (ACR) based on visualization of the graft tissue, lymphocytic infiltration, and vasculopathy. A scoring system was developed that assigned scores of either 1 (no evidence of pathology in question), 2 (cannot rule out evidence suspicious for pathology in question), 3 (strong evidence of pathology in question), or 0 (damaged tissue/insufficient tissue for analysis).

### Statistics

Experimental results were analyzed by GraphPad Prism software (GraphPad Software 10.0.3, San Diego, CA). All the data are presented as mean with individual values shown in figures. And compared using a non-parametric student's *t*-test with significance set at *p* less than 0.05. Survival curves were compared in GraphPad Prism with a Mantel-Cox Log-Rank test.

## Results

### Post-transplant immunoproteasome inhibitors does not change graft survival but may increase recipient mortality

Human CD52Tg mice received fully MHC mismatched C57BL/6 cardiac allograft. As shown in Figure 1A, heterotopic transplant recipients received peri-transplant alemtuzumab induction, which mediates T cell depletion and promotes long-term graft survival (21). Additional treatment of anti-CD25mAb (PC61 clone) has demonstrated accelerated AMR CAV development (24). This chronic AMR model typically does not promote cessation of heterotopic cardiac allografts (or acute rejection). Similarly, IPI group with additional post-transplant IPI treatment (alemtuzumab/PC61 induction with subsequent IPI 3 times weekly) did not change the graft survival compared to control (alemtuzumab/PC61 induction without maintenance immunosuppression) (Figure 1B). However, it is notable that there were more transplant recipients in the IPI group than the control group that died prior to study completion. One animal from each group died in the first post operative week and were subsequently censored from survival analysis. Of the remaining twenty mice assigned to the IPI group, twelve (60.0%) survived to end point compared to seven of the eight (87.5%) control mice. At the end of the study period, there was no significant difference in survival between the IPI and control groups (*p* = 0.1503).

### Post-transplant immunoproteasome inhibition suppresses post-transplant DSA production

**Figure 2:**
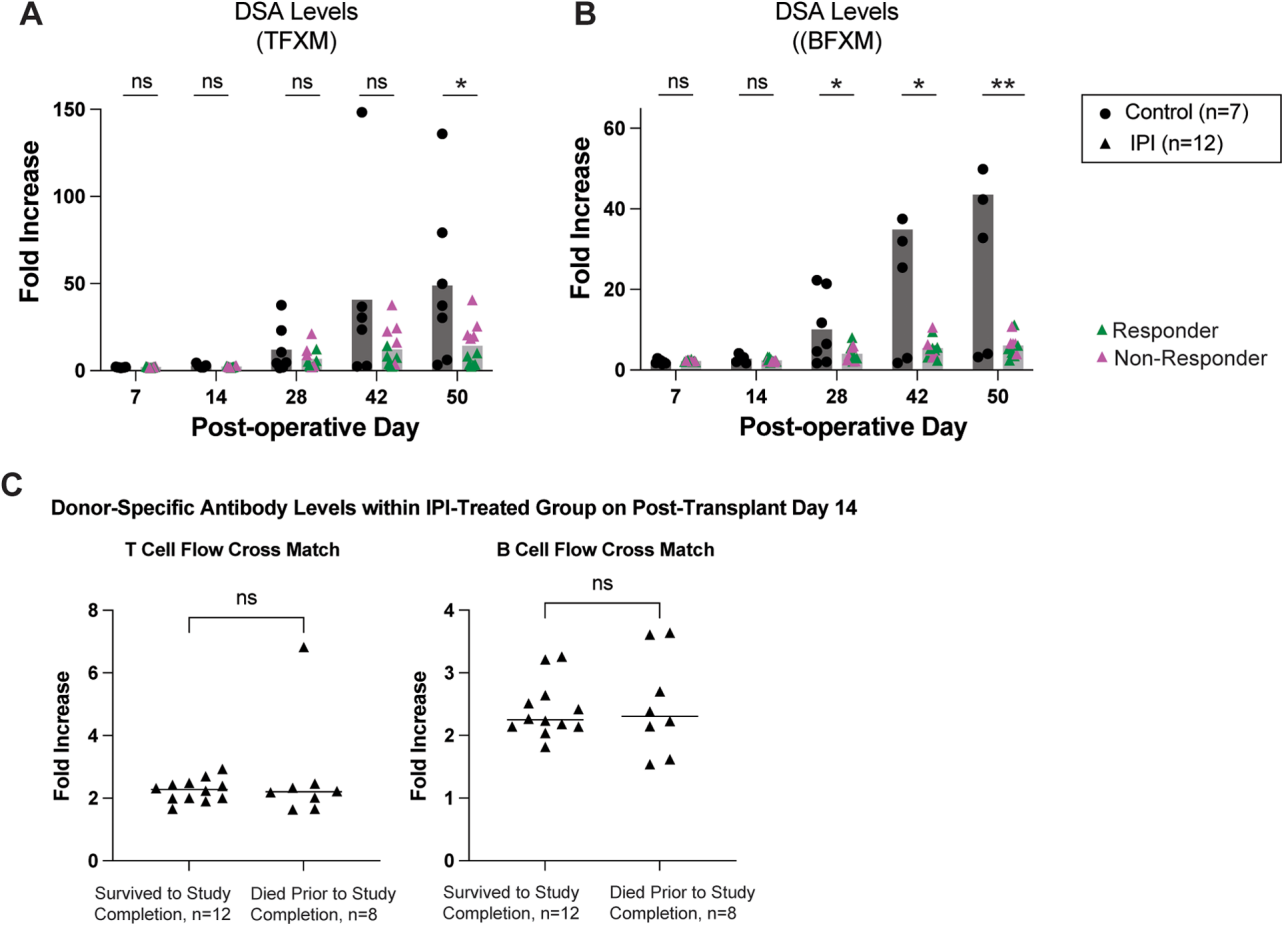
Survival in IPI-treated and control groups. **(A)** DSA production measured in TFXM. **(B)** BFXM throughout study period. **(C)** DSA levels in those that did not survive to study completion and those that died prematurely at day 14 in IPI group. Green triangles represent response to IPI treatment, pink represent nonresponse with DSA elevation. *N*, numbers indicate biologically independent animals; *p < 0.05; **P < 0.01 using two-tailed parametric paired *t*-test; NS, indicates no statistical significance.

Depletion of regulatory T cells with PC61 can accelerate development of donor-specific alloantibodies (24). As shown in Figure 2, the control group exhibited a gradual elevation of serum DSA. Interestingly, circulating DSA levels were noted to be significantly reduced in the IPI group for both TFXM (14.17 vs. 48.86-fold increase, *p* = 0.0291; Figure 2A) and BFXM (6.114 vs. 43.53-fold increase, *p* = 0.0031; Figure 2B) at study endpoint. BFXM was also significantly reduced in the IPI group at 4- and 6-weeks post-transplant (4.022 vs. 10.4, *p* = 0.0315 and 5.435 vs. 29.40, *p* = .0172, respectively; Figure 2B). Within the IPI group, five animals were considered non-responders based on DSA elevations as detailed above (Figure 2A).

To assess whether early mortality affected DSA production or vice versa among the 20 IPI-treated mice, we compared DSA levels between mice that died early (*n* = 8) and those that survived (*n* = 12) at post-operative day 14—the final timepoint at which sera were collected from all animals (Figure 2C). We detected no significant difference in DSA levels between the two groups as measured in a TFXM (*p* = 0.3110) or a BFXM (*p* = 0.5408).

### IPI treatment is associated with reduced histologic evidence of AMR

**Figure 3:**
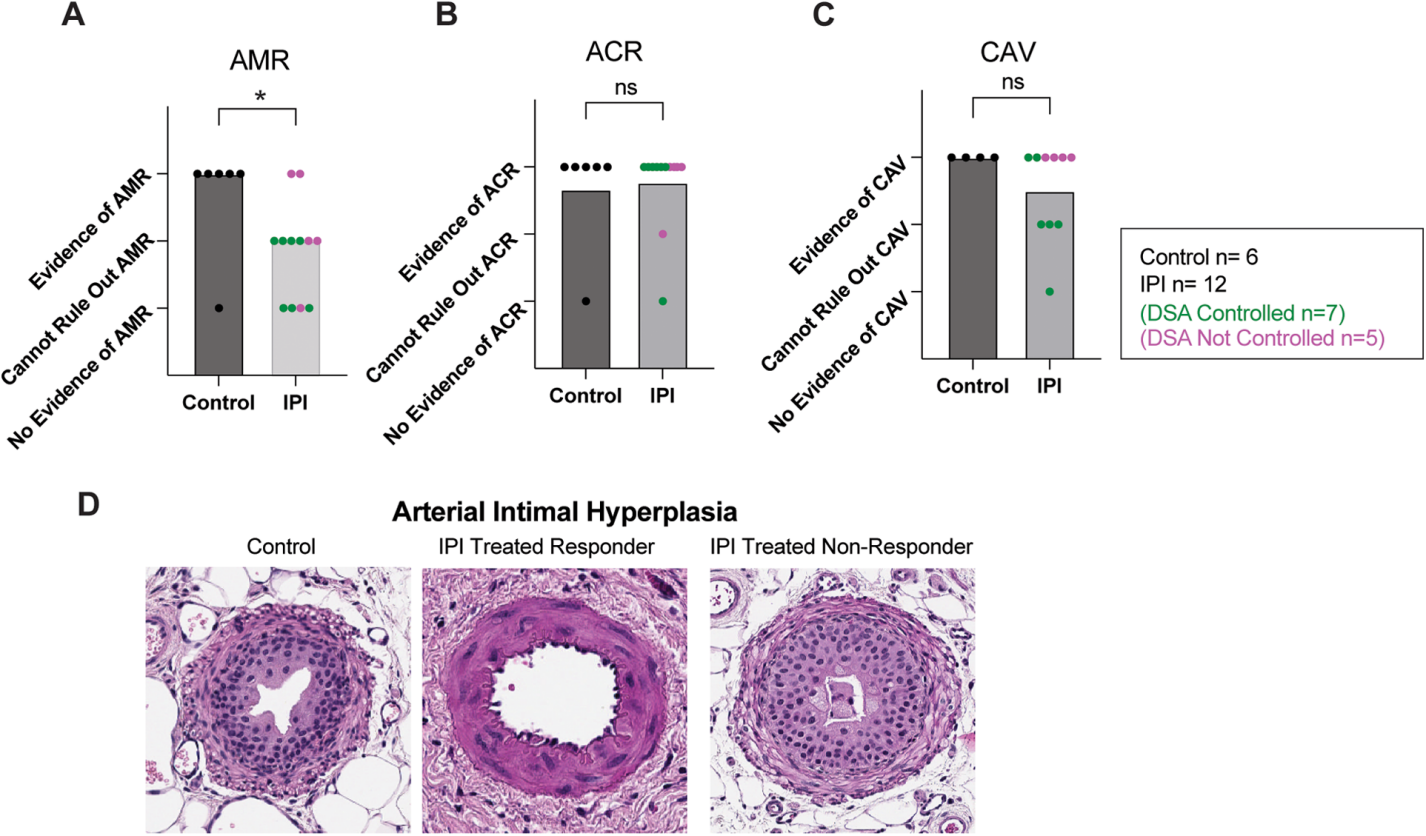
Histologic evidence of graft inflammation, damage, and rejection. **(A)** AMR scores compared between IPI and control groups, calculated as evidence of AMR = 3, evidence concerning for possible AMR = 2, and no evidence of AMR = 1. **(B)** ACR scores compared between IPI and control groups, calculated as evidence of ACR = 3, evidence concerning for possible ACR = 2, and no evidence of ACR = 1. **(C)** CAV scores compared between 9 IPI and 4 control grafts with sufficient vessels recovered for histologic evaluation, calculated as evidence of CAV = 3, evidence concerning for possible CAV = 2, and no evidence of CAV = 1. **(D)** Representative H&E images of arteries identified within control, IPI-treated responder, and IPI-treated non responder grafts. *N*, numbers indicate biologically independent animals; *p < 0.05 using two-tailed parametric paired *t*-test; NS, indicates no statistical significance.

Transplanted cardiac tissue was recovered for histological evaluation in six of seven controls and twelve of twelve IPI-treated animals surviving to study completion. Samples were deemed insufficient for CAV evaluation (score 0) in three of twelve in the IPI group and in two of six control group slides. Samples with insufficient visualization for evaluation were omitted from statistical analysis. Evidence of AMR was more prevalent in the control group than the IPI group (*p* = 0.0410, Figure 3A). Within the IPI group, evidence of AMR was observed in two of the five IPI-treated recipients deemed IPI-non-responders. There was no difference in incidence of ACR or CAV between control and IPI groups (*P* = 0.8118 and *P* = 0.2199, respectively) (Figures 3B,C).

Arterial Intimal hyperplasia was observed in both control and IPI-treated groups. Marked arterial hyperplasia was noted in an IPI-non-responder, while IPI-responders were found to have some non-hyperplastic vessels (Figure 3D).

## Discussion

In cardiac transplantation, AMR poses a threat to allograft survival through acceleration of graft dysfunction and development of coronary vasculopathy. While the pathogenesis is multifactorial (25), the development of DSA and subsequent episodes of AMR have been shown to correlate with progression of graft vasculopathy and atherosclerosis (6). Proteasome inhibition has emerged as a prominent strategy to target AMR across the field of transplantation (9, 15, 26). PIs induce apoptosis of antibody-producing plasma cells (27), and are used clinically to reduce DSA in episodes of acute AMR in solid organ transplant patients (28, 29). However, some studies have demonstrated that proteasome inhibition is inconsistently effective in managing AMR clinically (14, 30). Additionally, while plasma cells are particularly susceptible PIs due to high protein turnover rates, proteasomes are ubiquitous organelles, and indiscriminate inhibition leads to off-target effects. Common complications of proteasome inhibitors include cardiotoxicity, nephrotoxicity, anemia, and peripheral neuropathy (31–33). IPIs specifically target subunits of immunoproteasomes in cells of hematopoietic origin and those exposed to pro-inflammatory cytokines (34) instead of targeting constitutively expressed proteasomes in other cells. This selective inhibition has been shown to reduce toxicity profiles without compromising plasma cell depletion activity in multiple myeloma (18) and rat models of kidney transplant (19), however studies have yet to explore the impact of immunoproteasome inhibition on DSA production in heart transplantation and whether attenuating AMR subsequently reduces graft vasculopathy.

We have previously shown that alemtuzumab treatment promoted long-term graft survival in hCD52Tg recipients, however, these recipients developed *de novo* DSA, allo-B cells, and CAV (21). We also showed that this AMR phenotype can be accelerated or reversed with regulatory T cell depletion or co-stimulation blockade treatment, respectively (35). In the present study, we showed that IPI can attenuate DSA production, and the resultant AMR compared to the control group, but its effectiveness is potentially limited by toxicity and variable treatment responsiveness. Notably, there were three distinct phenotypes observed within the IPI-treated group: IPI response and reduction of DSA, IPI non-response with significant production of DSA, and presumed toxicity-mediated death. Among IPI-treated mice, approximately one-half demonstrated persistently controlled DSA through the study endpoint. These mice had correspondingly reduced incidence of AMR on pathology. CAV was also reduced in the IPI-treated group, however the difference between IPI and control groups was not statistically significant. Notably, some IPI-treated mice did not demonstrate persistently controlled DSA. This differential response has been also observed in multiple PI studies. In cardiac transplant recipients, nonresponse to carfilzomib and bortezomib have been observed clinically (30, 36). The Borteject trial also showed that proteasome inhibition did not perform better than placebo at reducing late antibody mediated rejection in kidney transplant recipients (14). Successful clinical and preclinical studies suggest that proteasome inhibition is most effective when used in combination with other therapies (27, 37, 38). Thus, IPI therapy may be similarly limited as monotherapy in our model. In multiple myeloma, IPI has also been used to enhance efficacy of constitutive proteasome inhibitors through feedback regulation (39). This approach may be possible in the transplant population during acute AMR, but it would increase the risk of toxicity.

A primary goal of immunoproteasome inhibition is reducing toxicity associated with proteasome inhibition through enhanced specificity. Multiple studies have reported improvements in toxicity and adverse effect profiles *in vivo* and *in vitro* with IPI compared to PI therapy (10, 20, 32). Recently, IPI demonstrated appropriate safety and tolerability in stage 2 clinical trials for dermatomyositis, despite several participants experiencing significant toxicity (40). Our study found that IPI treatment resulted in a high mortality rate. The premature deaths may be mediated by toxicity, as heterotopic heart transplants are nonfunctional, meaning graft rejection or failure would not affect overall survival. However, the possibility of human error due to more frequent handling cannot be ruled out, as control group animals did not receive placebo injections. The different level of tolerability to IPI may be due to varying levels of immunoproteasome expression in some animals. Post-transplant stress and inflammation could lead to widespread upregulation of immunoproteasomes in the setting of increased secretion of interferon gamma and other inflammatory cytokines (34), which could increase IPI toxicity. The observed heterogenous response to IPI has also been shown in cancer studies as well. Differential expression of proteasome subunits, increased expression of chaperone proteins, and elevated antioxidant concentrations have been shown to be protective against PI-mediated apoptosis (41, 42). Rapid proteasome adaptation (43) may contribute to the unresponsiveness to IPI. Alternating conventional proteasome inhibitors with IPI could help prevent proteasome adaptation and promote more effective plasma cell depletion. Future studies could investigate whether similar signatures predict IPI response in transplant recipients, and whether these could be tested for and utilized clinically.

This study has several limitations. A significant limitation was the unexpected high mortality in the treatment group during the study period. This could skew the recipient demographic, limiting the data to surviving animals. The causes of death in the treatment group were not clarified. Future studies should investigate potential toxicity and mortality and optimize dosing to minimize toxicity while maintaining the efficacy of IPI. Evaluation of CAV, neointimal hyperplasia, and their correlation to AMR was also limited by additional tissue damage. Inflammatory vascular damage in allografts develops through many pathways and is observed clinically with and without AMR (6, 36). While vasculopathy was observed in both IPI and control group grafts, ACR, stress, and inflammation may have contributed to observed inflammation, which may be mitigated with appropriate combination of IPI with other immunomodulatory therapies in the post-transplant period. Additionally, the impact of IPI on immune cell populations has not been fully elucidated. Therefore, future studies should also investigate how prolonged IPI therapy modulates B and T cell subpopulations to better understand its effects on immune responses and antibody production.

In our chronic AMR model, prolonged post-transplant IPI treatment reduced DSA production and AMR development, but it was associated potential toxicity and variable efficacy. The introduction of IPI as a more selective alternative capable of reducing humoral rejection, while expected to reduce toxicity compared to PI, may still cause significant off-target effects. Further characterization of IPI in large animal models, including nonhuman primates, will enhance our understanding of its safety and efficacy. This will improve the utility of IPI in transplantation settings and support its advancement toward clinical application.

## Data Availability

The original contributions presented in the study are included in the article, further inquiries can be directed to the corresponding author.

## References

[1] Wilhelm MJ. Long-term outcome following heart transplantation: current perspective. J Thorac Dis. (2015) 7(3):549–51. 10.3978/j.issn.2072-1439.2015.01.46 25922738 PMC4387387

[2] Ramzy D Rao V Brahm J Miriuka S Delgado D Ross HJ. Cardiac allograft vasculopathy: a review. Can J Surg. (2005) 48(4):319–27. 16149368 PMC3211528

[3] Khush KK Cherikh WS Chambers DC Harhay MO Hayes D Jr. Hsich E The international thoracic organ transplant registry of the international society for heart and lung transplantation: thirty-sixth adult heart transplantation report—2019; focus theme: donor and recipient size match. J Heart Lung Transplant. (2019) 38(10):1056–66. 10.1016/j.healun.2019.08.004 31548031 PMC6816343

[4] Mehra MR Raval NY. Metaanalysis of statins and survival in *de novo* cardiac transplantation. Transplant Proc. (2004) 36(5):1539–41. 10.1016/j.transproceed.2004.05.036 15251380

[5] Clerkin KJ Restaino SW Zorn E Vasilescu ER Marboe CC Mancini DM. The effect of timing and graft dysfunction on survival and cardiac allograft vasculopathy in antibody-mediated rejection. J Heart Lung Transplant. (2016) 35(9):1059–66. 10.1016/j.healun.2016.04.007 27423693 PMC5662939

[6] Loupy A Toquet C Rouvier P Beuscart T Bories MC Varnous S Late failing heart allografts: pathology of cardiac allograft vasculopathy and association with antibody-mediated rejection. Am J Transplant. (2016) 16(1):111–20. 10.1111/ajt.13529 26588356

[7] Russell ME Fujita M Masek MA Rowan RA Billingham ME. Cardiac graft vascular disease. Nonselective involvement of large and small vessels. Transplantation. (1993) 56(6):1599–601. 8279051

[8] Kfoury AG Hammond ME Snow GL Drakos SG Stehlik J Fisher PW Cardiovascular mortality among heart transplant recipients with asymptomatic antibody-mediated or stable mixed cellular and antibody-mediated rejection. J Heart Lung Transplant. (2009) 28(8):781–4. 10.1016/j.healun.2009.04.035 19632573

[9] Nguyen VP Kobashigawa JA. Antibody-medicated rejection after heart transplantation: diagnosis and clinical implications. Curr Opin Organ Transplant. (2020) 25(3):248–54. 10.1097/MOT.0000000000000754 32304428

[10] Neubert K Meister S Moser K Weisel F Maseda D Amann K The proteasome inhibitor bortezomib depletes plasma cells and protects mice with lupus-like disease from nephritis. Nat Med. (2008) 14(7):748–55. 10.1038/nm1763 18542049

[11] Kubiczkova L Pour L Sedlarikova L Hajek R Sevcikova S. Proteasome inhibitors—molecular basis and current perspectives in multiple myeloma. J Cell Mol Med. (2014) 18(6):947–61. 10.1111/jcmm.12279 24712303 PMC4508135

[12] Field-Smith A Morgan GJ Davies FE. Bortezomib (velcadetrade mark) in the treatment of multiple myeloma. Ther Clin Risk Manag. (2006) 2(3):271–9. 10.2147/tcrm.2006.2.3.271 18360602 PMC1936263

[13] Li J Basler M Alvarez G Brunner T Kirk CJ Groettrup M. Immunoproteasome inhibition prevents chronic antibody-mediated allograft rejection in renal transplantation. Kidney Int. (2018) 93(3):670–80. 10.1016/j.kint.2017.09.023 29229189

[14] Eskandary F Regele H Baumann L Bond G Kozakowski N Wahrmann M A randomized trial of bortezomib in late antibody-mediated kidney transplant rejection. J Am Soc Nephrol. (2018) 29(2):591–605. 10.1681/ASN.2017070818 29242250 PMC5791086

[15] Everly JJ Walsh RC Alloway RR Woodle ES. Proteasome inhibition for antibody-mediated rejection. Curr Opin Organ Transplant. (2009) 14(6):662–6. 10.1097/MOT.0b013e328330f304 19667989

[16] McCarthy MK Weinberg JB. The immunoproteasome and viral infection: a complex regulator of inflammation. Front Microbiol. (2015) 6:21. 10.3389/fmicb.2015.00021 25688236 PMC4310299

[17] Aki M Shimbara N Takashina M Akiyama K Kagawa S Tamura T Interferon-gamma induces different subunit organizations and functional diversity of proteasomes. J Biochem. (1994) 115(2):257–69. 10.1093/oxfordjournals.jbchem.a124327 8206875

[18] Nair N. Vascular rejection in cardiac allograft vasculopathy: impact on graft survival. Front Cardiovasc Med. (2022) 9:919036. 10.3389/fcvm.2022.919036 35990962 PMC9386065

[19] Li J Koerner J Basler M Brunner T Kirk CJ Groettrup M. Immunoproteasome inhibition induces plasma cell apoptosis and preserves kidney allografts by activating the unfolded protein response and suppressing plasma cell survival factors. Kidney Int. (2019) 95(3):611–23. 10.1016/j.kint.2018.10.022 30685098

[20] Karreci ES Fan H Uehara M Mihali AB Singh PK Kurdi AT Brief treatment with a highly selective immunoproteasome inhibitor promotes long-term cardiac allograft acceptance in mice. Proc Natl Acad Sci U S A. (2016) 113(52):E8425–32. 10.1073/pnas.1618548114 27956634 PMC5206568

[21] Kwun J Oh BC Gibby AC Ruhil R Lu VT Kim DW Patterns of *de novo* allo B cells and antibody formation in chronic cardiac allograft rejection after alemtuzumab treatment. Am J Transplant. (2012) 12(10):2641–51. 10.1111/j.1600-6143.2012.04181.x 22759336 PMC5464351

[22] Gilliland LK Walsh LA Frewin MR Wise MP Tone M Hale G Elimination of the immunogenicity of therapeutic antibodies. J Immunol. (1999) 162(6):3663–71. 10.4049/jimmunol.162.6.3663 10092828

[23] Kwun J Hu H Schadde E Roenneburg D Sullivan KA DeMartino J Altered distribution of H60 minor H antigen-specific CD8T cells and attenuated chronic vasculopathy in minor histocompatibility antigen mismatched heart transplantation in Cxcr3-/- mouse recipients. J Immunol. (2007) 179(12):8016–25. 10.4049/jimmunol.179.12.8016 18056341

[24] Oh B Yoon J Farris A 3rd Kirk A Knechtle S Kwun J. Rapamycin interferes with postdepletion regulatory T cell homeostasis and enhances DSA formation corrected by CTLA4-ig. Am J Transplant. (2016) 16(9):2612–23. 10.1111/ajt.13789 26990829

[25] Kwun J Knechtle SJ. Overcoming chronic rejection-can it B? Transplantation. (2009) 88(8):955–61. 10.1097/TP.0b013e3181b96646 19855237

[26] Basler M Li J Groettrup M. On the role of the immunoproteasome in transplant rejection. Immunogenetics. (2019) 71(3):263–71. 10.1007/s00251-018-1084-0 30220008

[27] Woodle ES Walsh RC Alloway RR Girnita A Brailey P. Proteasome inhibitor therapy for antibody-mediated rejection. Pediatr Transplant. (2011) 15(6):548–56. 10.1111/j.1399-3046.2011.01543.x 21884344

[28] Velleca A Shullo MA Dhital K Azeka E Colvin M DePasquale E The international society for heart and lung transplantation (ISHLT) guidelines for the care of heart transplant recipients. J Heart Lung Transplant. (2023) 42(5):e1–141. 10.1016/j.healun.2022.10.015 37080658

[29] Otero J Albers E Friedland-Little J Hong B Kemna M Gimferrer I Use of bortezomib in the treatment of antibody mediated rejection (AMR) in pediatric heart recipients. J Heart Lung Transplant. (2019) 38(4):S468–9. 10.1016/j.healun.2019.01.1192

[30] Horn ET Xu Q Dibridge JN Huston JH Hickey GW Kaczorowski DJ Reduction of HLA donor specific antibodies in heart transplant patients treated with proteasome inhibitors for antibody mediated rejection. Clin Transplant. (2023) 37(12):e15132. 10.1111/ctr.15132 37705362

[31] Fotiou D Roussou M Gakiopoulou C Psimenou E Gavriatopoulou M Migkou M Carfilzomib-associated renal toxicity is common and unpredictable: a comprehensive analysis of 114 multiple myeloma patients. Blood Cancer J. (2020) 10(11):109. 10.1038/s41408-020-00381-4 33149167 PMC7642386

[32] von Brzezinski L Saring P Landgraf P Cammann C Seifert U Dieterich DC. Low neurotoxicity of ONX-0914 supports the idea of specific immunoproteasome inhibition as a Side-effect-limiting. Therapeutic Strategy. Eur J Microbiol Immunol (Bp). (2017) 7(3):234–45. 10.1556/1886.2017.00025 29034113 PMC5632751

[33] Lataifeh AR Nusair A. Fatal pulmonary toxicity due to carfilzomib (kyprolis). J Oncol Pharm Pract. (2016) 22(5):720–4. 10.1177/1078155215588630 26044587

[34] Tubio-Santamaria N Ebstein F Heidel FH Kruger E. Immunoproteasome function in normal and malignant hematopoiesis. Cells. (2021) 10(7). 10.3390/cells10071577 34206607 PMC8305381

[35] Kwun J Park J Yi JS Farris AB Kirk AD Knechtle SJ. IL-21 biased alemtuzumab induced chronic antibody-mediated rejection is reversed by LFA-1 costimulation blockade. Front Immunol. (2018) 9:2323. 10.3389/fimmu.2018.02323 30374350 PMC6196291

[36] Horn ET Xu Q Tushak Z Binko M Dibridge JN Huston JH Significant reduction of donor specific antibodies in heart transplant recipients treated with proteasome inhibitors for antibody mediated rejection. J Heart Lung Transplant. (2022) 41(4):S410–1. 10.1016/j.healun.2022.01.1033

[37] Walsh RC Alloway RR Girnita AL Woodle ES. Proteasome inhibitor-based therapy for antibody-mediated rejection. Kidney Int. (2012) 81(11):1067–74. 10.1038/ki.2011.502 22336990

[38] Kwun J Burghuber C Manook M Ezekian B Park J Yoon J Successful desensitization with proteasome inhibition and costimulation blockade in sensitized nonhuman primates. Blood Adv. (2017) 1(24):2115–9. 10.1182/bloodadvances.2017010991 29296858 PMC5737135

[39] Kegyes D Gulei D Drula R Cenariu D Tigu B Dima D Proteasome inhibition in combination with immunotherapies: state-of-the-art in multiple myeloma. Blood Rev. (2023) 61:101100. 10.1016/j.blre.2023.101100 37291017

[40] Aggarwal R Goyal N Lam D Ng A Leff R Ray K Zetomipzomib demonstrates favorable long-term safety and tolerability profile without signs of immunosuppression: results from the PRESIDIO study and its open-label extension study in patients with dermatomyositis and polymyositis. American College of Rheumatology Convergence (2023).

[41] Mitra AK Harding T Mukherjee UK Jang JS Li Y HongZheng R A gene expression signature distinguishes innate response and resistance to proteasome inhibitors in multiple myeloma. Blood Cancer J. (2017) 7(6):e581. 10.1038/bcj.2017.56 28665416 PMC5520403

[42] McConkey DJ Zhu K. Mechanisms of proteasome inhibitor action and resistance in cancer. Drug Resist Updat. (2008) 11(4–5):164–79. 10.1016/j.drup.2008.08.002 18818117

[43] Woodle ES Tremblay S Brailey P Girnita A Alloway RR Aronow B Proteasomal adaptations underlying carfilzomib-resistance in human bone marrow plasma cells. Am J Transplant. (2020) 20(2):399–410. 10.1111/ajt.15634 31595669 PMC6984988

